# Causal evidence from invasive recordings reveals distributed acoustic feature encoding underlying categorical voice perception

**DOI:** 10.1016/j.neuroimage.2026.122064

**Published:** 2026-06-18

**Authors:** Jasmine L. Hect, Daniel A. Silliman, Kyle M. Rupp, Emily E. Harford, William P. Welch, Ruba Al-Ramadhani, Taylor J. Abel

**Affiliations:** a Department of Neurological Surgery, University of Pittsburgh Medical Center Children’s Hospital of Pittsburgh, Pittsburgh, Pennsylvania, United States; b Department of Pediatrics, University of Pittsburgh Medical Center Children’s Hospital of Pittsburgh, Pittsburgh, Pennsylvania, United States; c Department of Bioengineering, University of Pittsburgh, Pittsburgh, Pennsylvania, United States

**Keywords:** Voice perception, Auditory cortex, Lesion mapping, CCEPs, Tractography, Functional connectivity, Intracranial EEG

## Abstract

The superior temporal cortex has long been implicated in the perception of vocal sounds, yet most evidence remains correlational. Establishing causal necessity requires demonstrating that disruption of specific neural circuits produces selective perceptual deficits. Here, we report a pediatric patient who underwent intracranial EEG following resection of a left temporal pilocytic astrocytoma that removed anterior and middle superior temporal cortex but spared posteromedial Heschl’s gyrus (pmHG) and posterior superior temporal gyrus (pSTG). During an auditory task, broadband high-frequency (BHA) activity revealed voice-selective responses in pSTG, whereas pmHG showed robust auditory responses without category sensitivity. During a two-alternative forced-choice voice–nonvoice discrimination task, direct electrical stimulation of either pmHG or pSTG produced acute, reversible knockouts of categorization, whereas control-site stimulation did not. Cortico-cortical evoked potentials, Granger causality, and diffusion tractography demonstrated preserved bidirectional connectivity between pmHG and pSTG, indicating an intact posterior auditory circuit despite extensive anterior temporal loss. Together, these findings provide causal evidence that categorical voice perception depends on coordinated activity across posterior primary and nonprimary posterior auditory cortex.

## 1. Introduction

Humans recognize voices rapidly and automatically, supporting communication and social inference. Despite the robustness of this ability, its neural basis remains incompletely understood. Neuroimaging studies have consistently shown preferential activation to voices across anterior, middle, and posterior superior temporal gyrus (pSTG) and sulcus (pSTS), in responses to vocal sounds (Agus et al., 2017; Belin et al., 2000; Pernet et al., 2015; Rupp et al., 2022; Zhang et al., 2021). However, these findings remain largely correlational, leaving unresolved which auditory cortical representations are necessary for categorical voice perception (CVP), and whether apparent voice sensitivity reflects categorical processing per se or sensitivity to stimulus properties that covary with vocal sounds.

A central unresolved issue concerns the organization of voice processing within auditory cortex. Serial hierarchical models posit that CVP emerges through stepwise transformations from primary auditory cortex to higher-order temporal regions (Cohen et al., 2016; Morillon et al., 2022), predicting that early auditory areas provide prerequisite input to downstream voice-selective sites. In contrast, parallel-distributed models propose that primary and nonprimary auditory regions contribute jointly to categorical perception via partially independent, reciprocally connected pathways (Hamilton et al., 2021). Distinguishing the relative contributions of these accounts requires causal perturbation of specific auditory nodes while behavior is measured.

We tested whether CVP depends on a distributed posterior auditory circuit in which primary and nonprimary regions are jointly required. We present a patient with extensive resection of left STG and STS, with preservation of posteromedial Heschl’s gyrus (pmHG) and pSTG. This lesion pattern constrains auditory processing in the left hemisphere eliminating large portions of canonical "temporal voice areas”. We combined quantitative lesion mapping, intracranial recordings, focal electrical stimulation, and connectivity analyses to evaluate the causal contributions of pmHG and pSTG to CVP. If CVP depends on a distributed encoding in a posterior auditory circuit, then perturbation of either node would impair CVP.

## 2. Results

### 2.1. Case description

The patient was a right-handed female who underwent surgical resection at age three for a left thalamic pilocytic astrocytoma extending into the anterior temporal lobe. Postoperatively, she recovered with right hemiparesis and demonstrated age-appropriate language development aside from mild dysfluency of speech characterized by partial and whole word repetition, with occasional blocking and sound prolongation. Over subsequent years, however, she developed persistent interictal epileptiform discharges localized to her residual left temporal pole, accompanied by intermittent cognitive slowing, gradual decline in IQ scores, and worsening severity and frequency of speech dysfluency. At age 9, she underwent intracranial electroencephalography (iEEG) for seizure localization as part of her clinical evaluation.

**Fig. 1.**
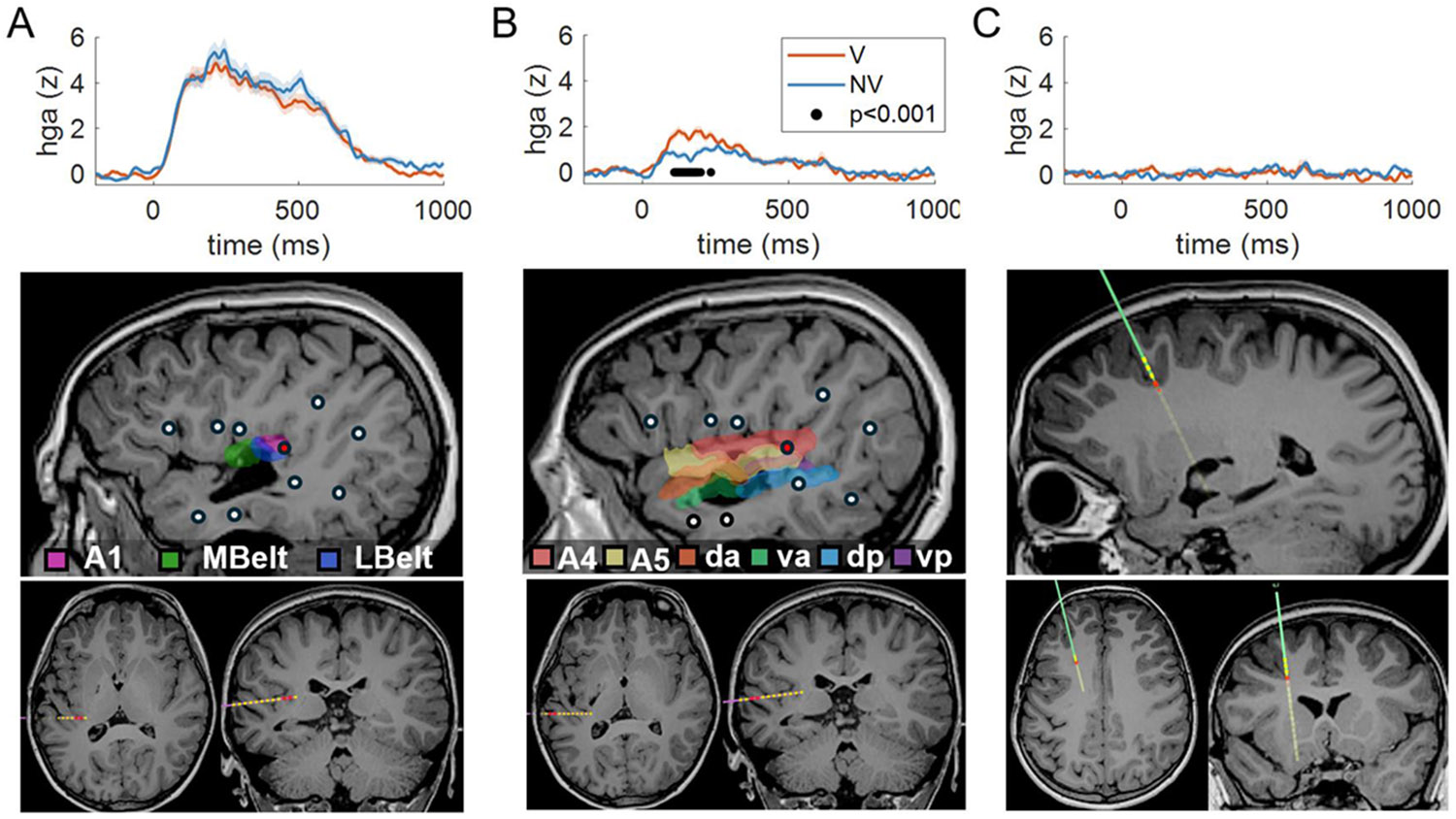
Identification of primary and nonprimary auditory sites for causal testing of voice perception. **(A–C)** Z-scored broadband high-frequency activity (BHA; 70–150 Hz) recorded with iEEG during an auditory n-back task). Red traces denote mean BHA to vocal (V) sounds; blue traces denote non-vocal (NV) sounds; shading indicates ± SEM across trials. Black bars mark time windows with significant V > NV separability (cluster-based permutation *t*-test, *p* < 0.001). **(A)** pmHG (A1) showed robust auditory responses without category sensitivity. **(B)** pSTG (A4) exhibited strong voice-selective responses. **(C)** A superior frontal sulcus (SFS) site served as a non-auditory control. Anatomical panels show electrode locations relative to the resection cavity, with auditory regions of interest (ROIs) defined using the extended Human Connectome Project atlas (A4/A5 = STG; da/va = dorsal and ventral anterior superior temporal sulcus (STS); dp/vp = dorsal and ventral posterior STS. Red markers indicate the bipolar iEEG depth electrode pair; white markers denote additional recording coverage.

Pre-implant neuropsychological assessment revealed intact hearing thresholds, normal speech perception, and preserved naming abilities. Structural MRI demonstrated extensive resection of anterior and middle STG and STS, with sparing of pSTG and pmHG (Fig. 1A-B). Quantitative lesion mapping of the resection cavity against a functional parcellation (Human Connectome Project) localized the resection to temporal regions. This procedure estimated the residual volume of auditory areas across regions of interest (ROIs): A5 50%; ventral and dorsal anterior STS 19%; auditory belt 41% and parabelt cortices 14%; para-insular cortex 50%), while pmHG (A1) STG (A4), and pSTS were largely intact (>85% (Fig. 1). This lesion configuration provided a rare opportunity to examine auditory processing within a constrained cortical network.

During research testing, the patient performed an auditory n-back working memory task and an orthogonal two-alternative forced-choice voice–nonvoice categorization task while undergoing intracranial recording and focal electrical stimulation. This experimental design enabled direct assessment of the functional and causal contributions of intact posterior auditory cortex to human CVP.

### 2.2. Primary and nonprimary auditory cortices show coactivation, causal interaction, and dissociable voice sensitivity tuning

Quantitative lesion mapping based on preoperative MRI used for iEEG trajectory planning confirmed extensive resection of anterior and middle temporal regions (A5 ≈ 50%; dorsal and ventral STS ≈ 81%; auditory belt ≈ 59%; parabelt cortex ≈ 86%; para-insular cortex ≈ 50%), while posteromedial Heschl’s gyrus (pmHG; A1) and posterolateral superior temporal gyrus (STG; A4) were largely spared (< 15%; Fig. 1). This anatomical constraint effectively limited the candidate substrates for residual categorical voice perception to posterior auditory cortex, motivating targeted functional and causal interrogation of pmHG and pSTG. To identify candidate sites for electrical stimulation, we first localized cortical regions exhibiting differential responses to vocal (V) and non-vocal (NV) sounds during an auditory one-back listening task (Voice Localizer; Fig. 1). Broadband high-frequency activity (BHA; 70–150 Hz) was used as a proxy for local population-level neuronal activity and quantified as z-scored event-related responses. Using cluster-based permutation testing on time-resolved t-statistics, we identified a channel pair in pSTG that was robustly auditory-responsive (54–499 ms, *p* < 10^−3^) and exhibited significant voice sensitivity (V > NV BHA; 109–234 ms, *p* < 10^−3^). In contrast, a channel pair in posteromedial Heschl’s gyrus (pmHG) showed strong, sustained auditory responses (39–499 ms, *p* < 10^−3^) without category sensitivity. A third site in superior frontal sulcus (SFS) showed no significant task-evoked responses and was therefore selected as a non-auditory comparison region for subsequent stimulation experiments. Although multiple sites lacked auditory responsiveness, SFS was chosen based on clinical considerations indicating a low risk of after-discharge during stimulation.

We next compared the temporal dynamics of auditory responses across sites. Single-trial latency analyses revealed no significant differences in BHA onset timing between pmHG and pSTG, either across all stimuli (t = −1.38, *p* = 0.17) or within vocal (t = −1.41, *p* = 0.16) or non-vocal (t = −0.54, *p* = 0.59) conditions.

**Fig. 2.**
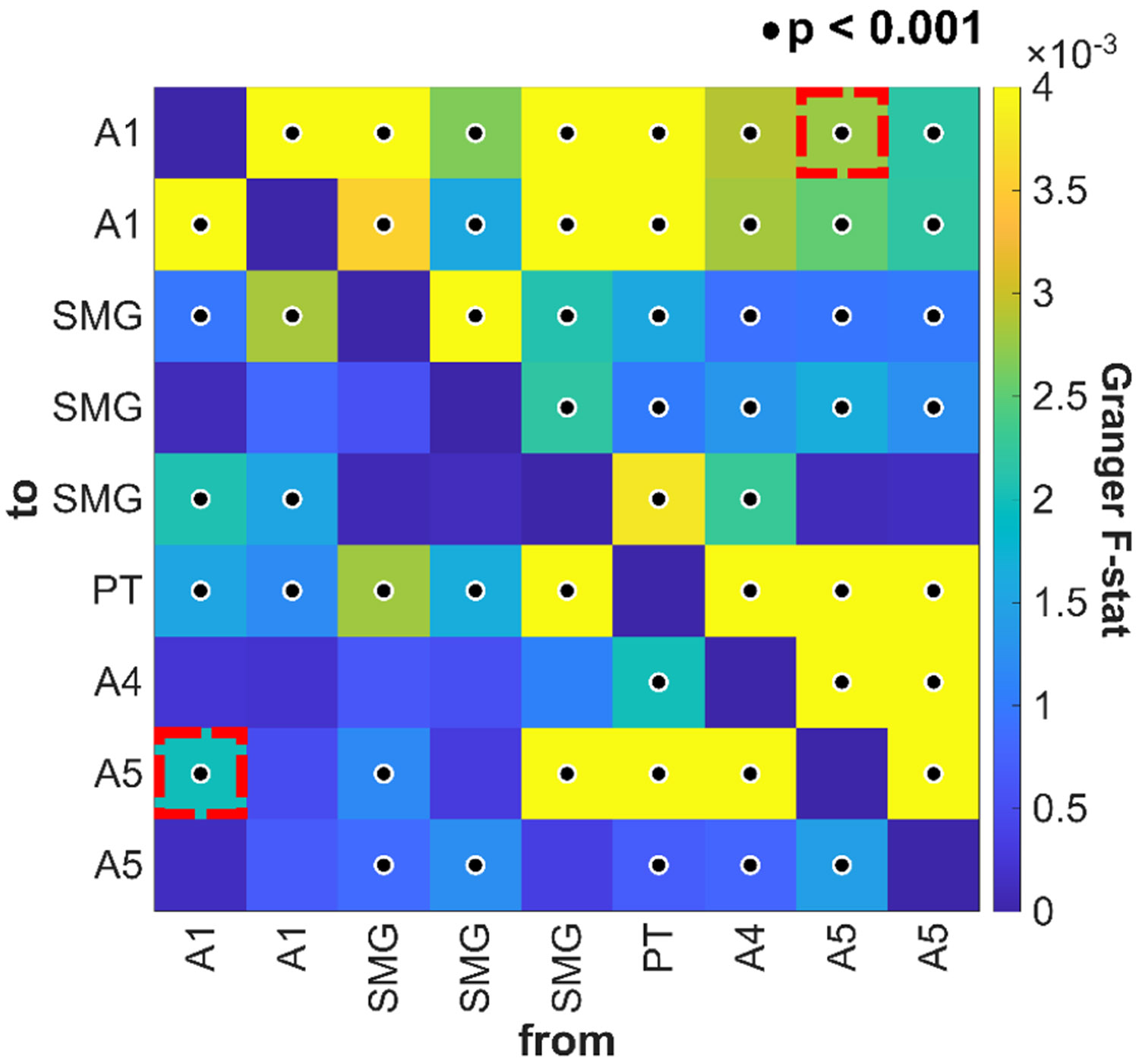
Multivariate granger causality (MVGC) during auditory n-back task. Time-domain MVGC analysis estimated significant (*p* < 0.001, FDR-corrected) Granger-causal interactions between pmHG and pSTG channel pairs; red highlighted cells indicate bidirectional coupling at the sites in pmHG and pSTG which were identified as primary auditory and voice-sensitive in BHA analysis during Voice Localizer, and which were also the stimulation targets during the behavioral and CCEP experiments (see below). A1 = core auditory cortex on pmHG, SMG = supramarginal gyrus, PT = planum temporale, A4 = auditory association complex 4 on pSTG, A5 = auditory association complex 5 on pSTG.

Finally, we used time-domain state-space multivariate Granger causality (MVGC) to test directed informational exchange during auditory perception (Fig. 2), which found bidirectional connections between pmHG and pSTG that were statistically significant (*p* < 0.001, FDR-corrected). Supramarginal gyrus (SMG) also exhibited significant bidirectional coupling with core and auditory association areas (*p* < 0.001, FDR-corrected).

### 2.3. Stimulation of posterior auditory cortices disrupts categorical voice perception

**Fig. 3.**
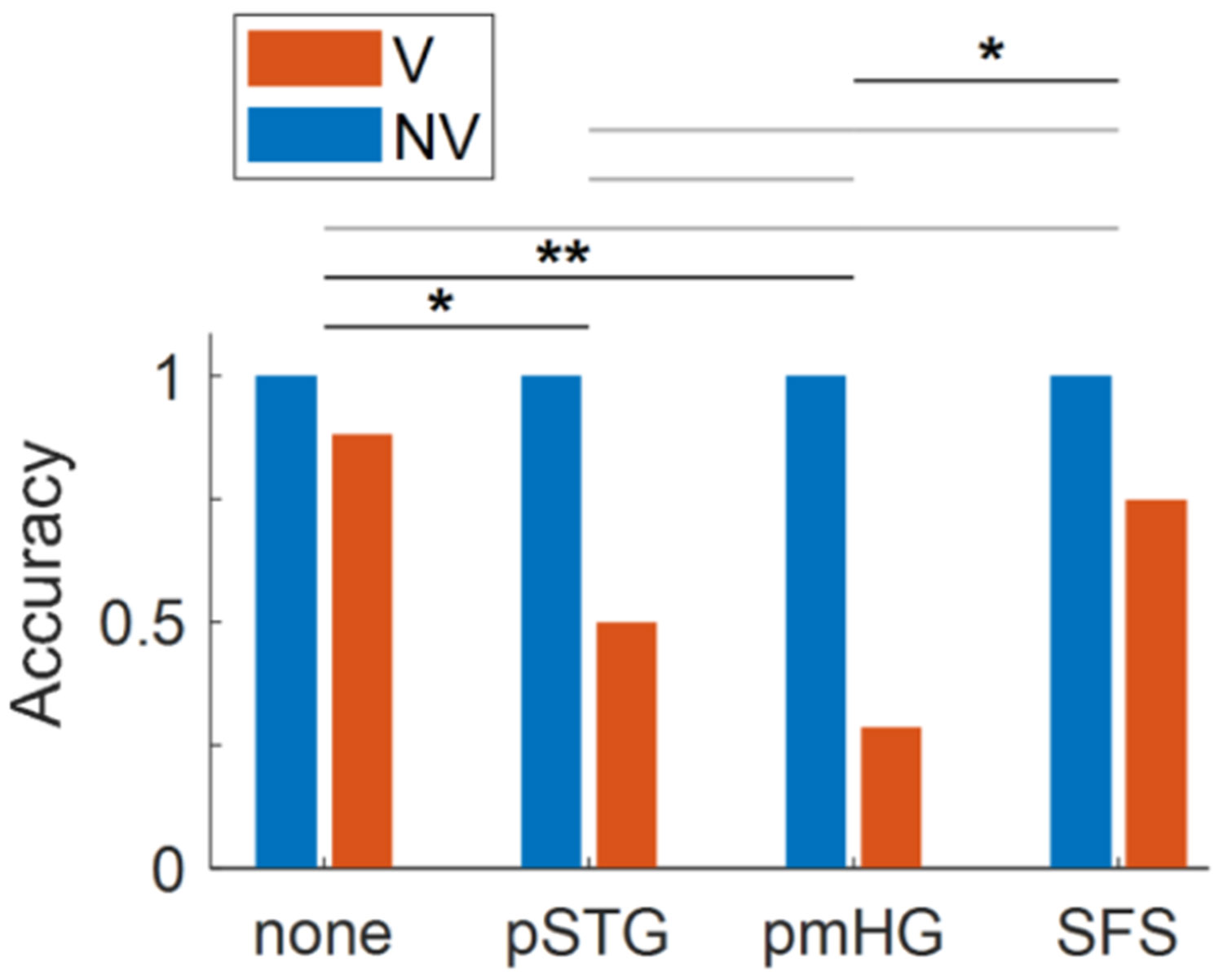
Disruption of categorical voice perception (CVP) during stimulation of posterior auditory cortex. Behavioral accuracy during a two-alternative forced-choice categorical voice perception (CVP) task under four stimulation conditions: sham (none), posterior superior temporal gyrus (pSTG), posteromedial Heschl’s gyrus (pmHG), and superior frontal sulcus (SFS, control). Bars show proportion correct for vocal (V) and non-vocal (NV) trials. Low-frequency (1 Hz) bipolar electrical stimulation of either pSTG or pmHG significantly reduced categorization accuracy relative to sham (none) and comparison (SFS) stimulation conditions. Horizontal lines indicate pairwise Fisher’s exact test comparisons (FDR-corrected; *p < 0.05; **p < 0.01).

To test whether activity in preserved posterior auditory regions is causally necessary for CVP in this patient, we administered low-frequency (1 Hz) bipolar electrocortical stimulation (2 mA, 25 ms pulses, biphasic) in concert with stimulus presentation during three of four total conditions while the subject completed a V-NV category discrimination task. The stimulation sites included a voice-sensitive site in pSTG, an auditory site agnostic to voice category in pmHG, a non-auditory comparison site in SFS.The fourth condition was completed the same by the patient, but no stimulation was applied (Fig. 3).

Stimulation of posterior auditory cortex produced site-specific and reversible impairments in CVP performance. Deficits were confined to trials during which stimulation was delivered, with no evidence of sustained performance degradation across the testing session. In contrast, stimulation of pmHG significantly reduced categorization accuracy relative to both sham (*p* < 0.01) and SFS comparison (*p* < 0.05). Stimulation of pSTG produced a comparable reduction in accuracy relative to control conditions. This dissociation suggests that the disruption was not attributable to general stimulation effects but was specific to auditory cortical perturbation. The magnitude of the behavioral deficit did not differ significantly between pmHG and pSTG stimulation (*p* = 0.28; Fig. 3). Performance recovered to baseline (sham condition) levels during stimulation of SFS relative to sham (*p* = 0.40), confirming that non-auditory stimulation did not significantly disrupt task performance. Together, results demonstrate that both pmHG and pSTG are causally necessary for accurate voice–nonvoice categorization. However, because pmHG and pSTG are densely interconnected, the observed behavioral deficits during pmHG stimulation could also reflect indirect disruption of pSTG-mediated categorization rather than an independent categorical role of pmHG itself.

### 2.4. Bidirectional functional coupling between pmHG and pSTG

Low-frequency (1 Hz) bipolar biphasic stimulation of either pmHG or pSTG was sufficient to abolish CVP, suggesting that disruption at either node perturbs a shared functional circuit. To probe the network mechanism underlying this behavioral effect, we examined effective connectivity between pmHG and pSTG using CCEPs elicited by the same stimulation paradigm.

**Fig. 4.**
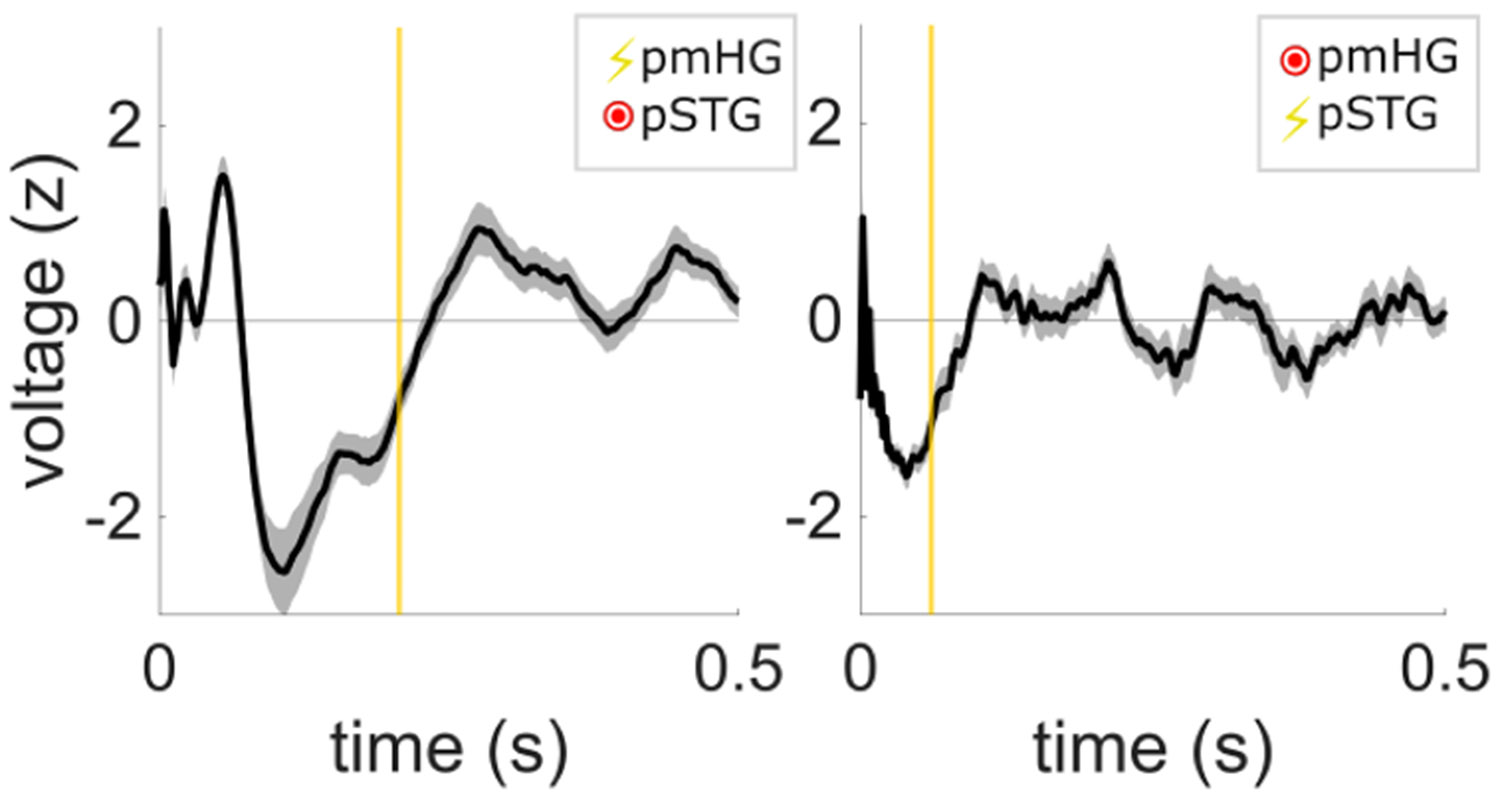
Bidirectional effective connectivity between spared posterior auditory regions. CCEPs recorded in pSTG during stimulation of pmHG (left) and in pmHG during stimulation of pSTG (right). Black traces show the mean evoked response ± SEM across artifact-free trials Vertical yellow lines indicate the duration of the canonical response, defined as the interval over which the evoked signal reliably deviated from baseline. For response parameterization the first 15 ms following stimulation were excluded to minimize contamination by stimulation artifact. Both stimulation–recording pairs produced significant evoked responses (*p* < 10^−3^, FDR-corrected), with distinct response morphologies.

We quantified CCEPs using canonical response parameterization (CRP),(Miller et al., 2023) which enables artifact rejection, estimation of the duration of reliable evoked activity, and statistical testing against a null baseline. Significant evoked responses were observed in pSTG during stimulation of pmHG and in pmHG during stimulation of pSTG (both *p* < 10^−3^, FDR-corrected; Fig. 4). No significant responses were detected for control-site stimulation at SFS ↔ pmHG/pSTG, indicating anatomical specificity.

The canonical response in pSTG following pmHG stimulation was prolonged (≈ 209 ms) and biphasic, with an early positive peak at 54 ms and a later negative peak at 107 ms. In contrast, pmHG responses to pSTG stimulation were shorter (63 ms) and monophasic, peaking at 40 ms. Despite these differences in temporal profile, response magnitude (quantified as the normalized weight of the first principal component across trials) was significantly greater for pmHG → pSTG and pSTG → pmHG than for any pair involving SFS (all *p* < 10^−3^). Response magnitude and signal-to-noise ratio (SNR) did not differ between the two auditory directions, consistent with symmetrical bidirectional coupling.

**Fig. 5.**
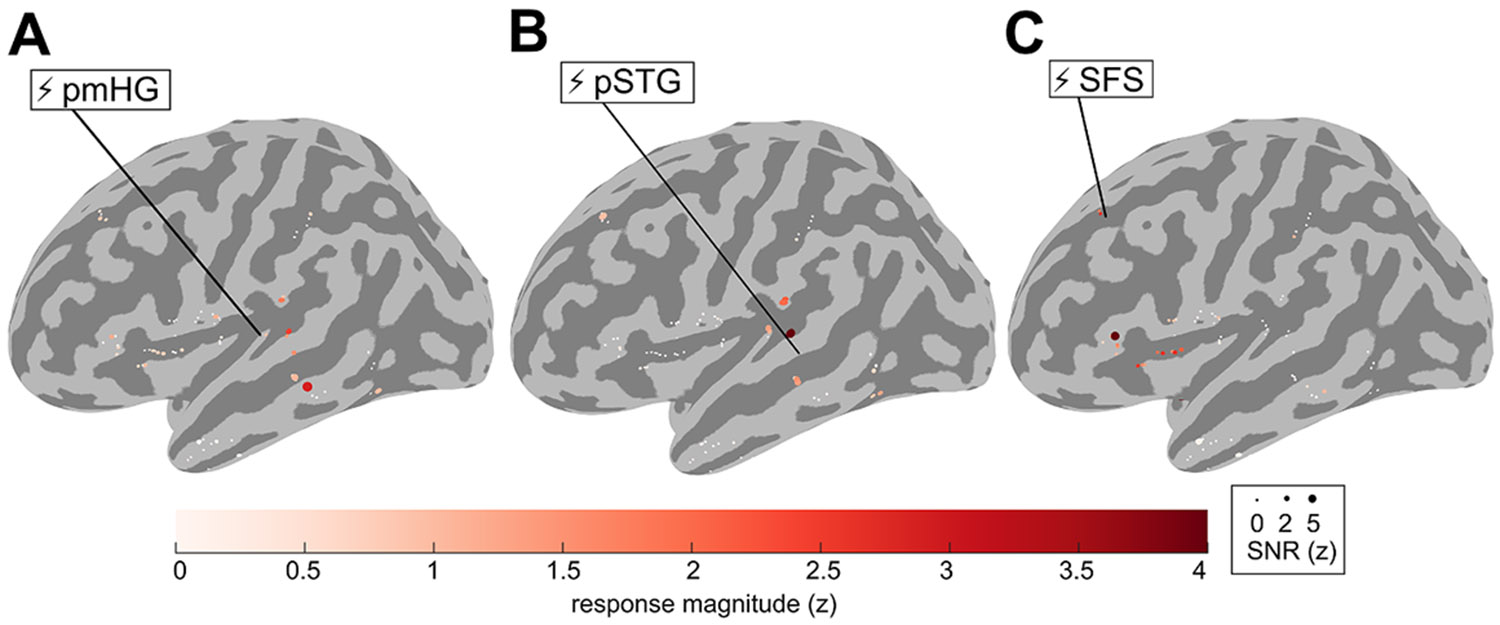
CCEPs during each of the three stimulation conditions in all valid channel pairs. Response magnitude corresponds to color, signal-to-noise ratio (SNR) to size. (A) During stimulation of pmHG, diffuse high-magnitude, high-SNR responses were observed in posterior superior temporal cortices. (B) Responses during stimulation of pSTG were similar to pmHG. (C) During stimulation of SFS, frontal opercular regions including Brodmann Area 45 showed high-magnitude, high SNR-responses.

**Fig. 6.**
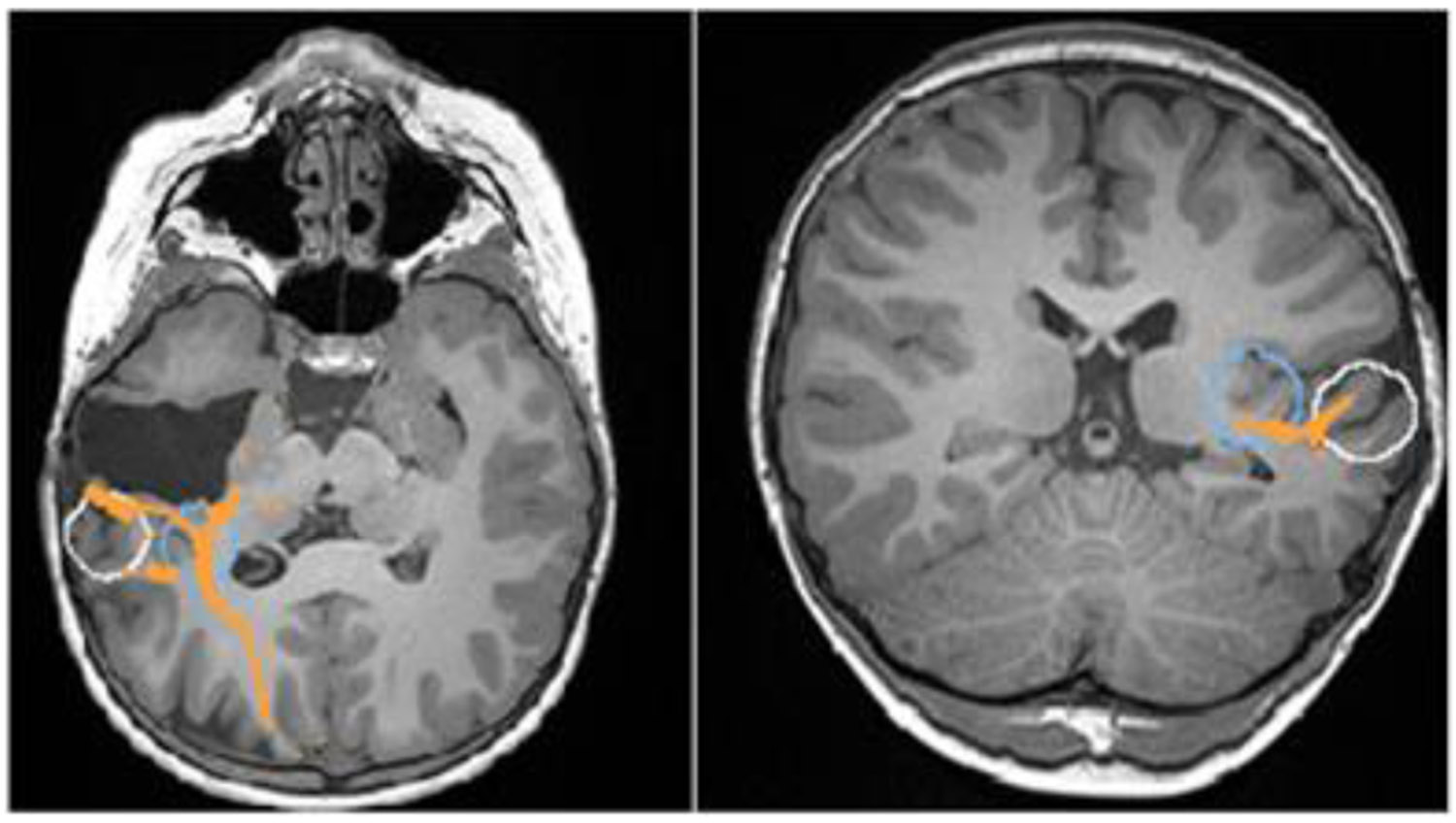
Structural connectivity between pmHG and pSTG. Deterministic diffusion tractography demonstrating direct white-matter connections between pmHG (blue circle) and pSTG (white circle) within the spared posterior temporal lobe. Streamlines (orange) visualize structural connectivity consistent with the bidirectional effective connectivity observed in MVGC and CCEPs analyses.

To supplement our analysis of bidirectional functional connectivity of the three stimulation-recording pairs, we calculated the magnitude and SNR of CCEPs in all other valid channel pairs during the three stimulation conditions (Fig. 5). During stimulation of both pmHG and pSTG, high-magnitude, high-SNR responses were observed in pSTG and pSTS, as well as the inferior supramarginal gyrus (SMG), which was convergent with our MVGC findings. During stimulation of SFS, high-magnitude, high-SNR responses were clustered around frontal opercular regions, including Brodmann Area 45. Finally, to assess the structural substrates supporting interactions between pmHG and pSTG in particular, we performed deterministic fiber tractography (Yeh, 2025; Yeh et al., 2013). Tractography revealed direct white-matter pathways linking pmHG and pSTG (Fig. 6). Together, functional and structural findings indicate that pmHG and pSTG form a reciprocally connected posterior auditory subnetwork in this patient, providing a mechanistic substrate for the equivalent behavioral knockouts observed during stimulation of either site.

## 3. Discussion

Categorical perception of vocal sounds is a fundamental ability of human auditory cognition, yet the neural mechanisms that support it remain incompletely resolved. In this study, we leveraged a rare combination of extensive temporal lobe resection, intracranial recordings, focal electrical stimulation, and connectivity analyses to identify the minimal posterior auditory circuitry necessary for CVP. The patient’s lesion encompassed anterior and middle STG and STS, effectively restricting residual auditory processing to posterior temporal cortex. pmHG exhibited strong auditory responses without voice category sensitivity, consistent with encoding spectrotemporal features common to many sound classes (Brugge et al., 2009; Gnanateja et al., 2025; Howard et al., 1996; Khalighinejad et al., 2021; Langers and van Dijk, 2012). In contrast, pSTG showed response sensitivity to vocal sounds compared to non-vocal sounds, supporting its role in the spared left hemisphere “voice areas”(Belin et al., 2000;Bodin et al., 2021). Within this constrained network, stimulation of either pmHG or pSTG produced acute and reversible impairments in performance on a voice–nonvoice categorization task, whereas stimulation of a non-auditory control site in the frontal lobe had no effect.

Analysis of BHA response dynamics during the baseline auditory working memory n-back task supports distributed feature encoding within the left posterior auditory cortex. First, BHA trial-wise response latency onsets did not differ significantly between pmHG and pSTG, suggesting both regions are engaged on comparable timescales following sound onset. Response onset did not demonstrate sensitivity to vocal category, indicating that the pattern of voice sensitivity in pSTG emerges over time, in concert with lower-level acoustic analysis in pmHG (Giordano et al., 2023; Rupp et al., 2025; Staib and Frühholz, 2022). Second, evidence of bidirectional communication between pmHG and pSTG during auditory perception is provided by MVGC analysis. Together, this suggests pmHG and pSTG demonstrate rapid and recurrent communication during listening. In this view, the communication of complex acoustic features between regions is necessary for categorical judgments of voiceness.

The stimulation findings demonstrate that disruption of either node is sufficient to impair the circuit-level computation supporting CVP. Given that stimulation of either site can propagate to the other through preserved white-matter connections, the stimulation results are most appropriately interpreted as establishing the causal necessity of the posterior auditory circuit as a network, rather than isolating independent causal roles for each node. What the data do rule out is a model in which pSTG is dispensable for CVP, or in which posterior auditory regions operate independently. Of note, the use of low-frequency (1 Hz) electrocortical stimulation was intended to keep functional disruption as focal to the regions of interest as possible (Mohan et al., 2020). Therefore, CVP appears to depend on the integrity of a tightly coupled posterior temporal network, whether computations are localized to pSTG or emerge through recurrent interactions across posterior auditory cortex.

Both effective and structural connectivity analyses revealed strong bidirectional coupling between pmHG and pSTG, consistent with prior CCEP–DTI analyses in the language system (Conner et al., 2011; Ookawa et al., 2017). CCEPs demonstrated significant evoked responses in both directions, with distinct but complementary morphologies, while diffusion tractography identified direct white-matter pathways linking pmHG and pSTG. In the context of latency and Granger-causal modeling results, these reciprocal connections likely support coordination and stabilization of distributed acoustic feature representations across posterior auditory cortex. Such interactions may allow feature representations encoded at different spatial and temporal scales to be jointly evaluated during perceptual decision-making. The CCEP morphologies seen in pmHG and pSTG were consistent with prior reports (Trébuchon et al., 2021) and also similarly demonstrated a large early negative component characteristic of auditory evoked potentials. It is relevant to note that in our case, these similarities were not related to any perceptual correlate, given our stimulation protocol first finds the maximum safest amplitude without any somatosensory response. Whether these grossly similar morphologies in CCEPs in AEPs reflect a common physiologic basis is an interesting point to consider in future work. Taken together, the results presented here provide multimodal, causal evidence for a posterior auditory network capable of supporting CVP independent of canonical anterior and middle temporal “voice areas”.

In the intact brain, anterior and middle STG/STS likely contribute additional acoustic, contextual, or integrative information that refines voice perception, supports robustness across listening conditions, or enables more complex vocal judgments beyond binary categorization (Ethofer et al., 2012; Giamundo et al., 2025). Their absence in this patient may therefore place increased computational demands on posterior auditory regions, rendering both pmHG and pSTG jointly critical for maintaining sufficient acoustic evidence to support CVP. While the behavioral knockouts produced by left-hemisphere stimulation demonstrate the causal necessity of the left posterior auditory circuit for task performance, regardless of any potential right-hemisphere contribution, the contribution of the spared posterior auditory network in the right-hemisphere cannot be excluded in this patient. Previous work showed that repetitive transcranial magnetic stimulation to right hemisphere temporal “voice areas” produced deficits in CVP (Bestelmeyer et al., 2011). Although our patient’s electrode coverage did not permit perturbations to right auditory cortex, the behavioral knockouts observed here and in prior work indicate that when this posterior circuit is disrupted, no alternative temporal pathway remained capable of compensating, highlighting the necessity of distributed acoustic feature encoding across posterior auditory cortex.

Several factors may limit generalizability of these findings and warrant additional consideration. First, causal inference is inherently constrained by the single-case design and the patient’s atypical anatomy following early temporal resection. In future work, comparison to aged-matched controls in the categorization task may help elucidate the contribution of the resected anterior temporal regions to CVP. At present, we cannot conclude whether the resected temporal regions would contribute to categorization in healthy age-matched controls.

Second, further investigation should consider extratemporal contributions to CVP which could not be ruled out due to the sparse cortical coverage of iEEG in this case. Effective connectivity analyses did identify channel pairs within the ventral bank of SMG, a site that may serve an important role in CVP or auditory processing more broadly. Both CCEPs and MVGC showed that this region received significant input from both pmHG and pSTG during stimulation and during the n-back task. The apparent salience of SMG to auditory working memory may be relevant to an interpretation of these results (Lerud et al., 2021); however, because we did not stimulate SMG directly, we cannot ascertain its specific causal role, if any, in CVP. Replication in larger cohorts utilizing a more exhaustive stimulation protocol will be necessary to establish the generality of our findings.

Third, although bipolar re-referencing minimized stimulation artifacts and no after-discharges were detected, transient spiking observed in some channel pairs immediately following stimulation may reflect local hyperexcitability or network-level propagation. In addition, early developmental plasticity may have contributed to reorganization within the intact cortex, potentially enhancing its capacity to support CVP in the absence of anterior superior temporal regions. Fourth, because the behavioral CVP paradigm required binary voice–nonvoice judgments, it cannot distinguish whether stimulation-induced deficits reflect disruption of category boundary formation or a more general impairment in auditory decision-making. Future studies incorporating multi-class categorization, graded confidence measures, or reaction-time analyses may help dissociate these possibilities and more precisely characterize the acoustic feature dimensions that support voice perception.

## 4. Conclusion

This study provides convergent causal evidence that CVP depends on distributed acoustic feature encoding across posterior auditory cortex. In a patient with extensive resection of anterior and middle temporal regions, stimulation of either pmHG or pSTG abolished voice–nonvoice categorical discrimination, despite preserved structural and functional connectivity between these sites. Beyond refining theoretical models of auditory cortical organization, these findings highlight the functional importance of posterior auditory circuitry for voice perception and underscore the need to preserve connectivity between primary and nonprimary auditory regions during temporal lobe resections. More broadly, this work illustrates how invasive neurophysiology in and lesion mapping can reveal the minimal neural substrates necessary for complex perceptual functions.

## 5. Methods

### 5.1. Electrode implantation, recording, and localization

Written informed consent and assent for research was obtained from the patient, and the research protocol was approved by the University of Pittsburgh Institutional Review Board (STUDY19010005). The patient completed preoperative neuropsychological evaluation. As part of a clinical evaluation for epilepsy surgery, iEEG electrodes were implanted in the patient’s left temporal lobe, using techniques described in prior work (Abel et al., 2018) Dixi Medical Microdeep electrodes were used, with a diameter of 0.8 mm, contact length of 2 mm, and center-to-center spacing of 3.5 mm.

### 5.2. Intracranial recordings and broadband high-gamma extraction

Neural data were recorded using the Ripple Grapevine Nomad system (model R02000) at a sampling rate of 1 kHz. Neural data during stimulation experiment was recorded using the Natus Quantum system at a sampling rate of 512 Hz. Auditory stimuli were presented binaurally via Etymotic ER-3C insert earphones. To achieve precise temporal synchronization between the auditory and neural data streams, the play-back signal was simultaneously routed through a high-resolution auxiliary analog input, recorded at 30 kHz through a Ripple Digital/Analog IO box. This allowed us to directly monitor the acoustic waveform as it was delivered to the participant’s ears. Digital triggers marking stimulus onset, offset, and behavioral responses were also recorded using a National Instruments Data Acquisition system and logged alongside neural data via the same IO interface. This multimodal recording setup enabled millisecond-level alignment between stimulus presentation and neural activity.

Preprocessing of the neural data involved several steps to ensure data quality. First, raw signals were bandpass filtered from 1 to 200 Hz to retain the relevant physiological frequency range, and notch filters at 60, 120, and 180 Hz were applied to eliminate electrical line noise. Channels with excessive broadband noise were excluded based on a robust thresholding criterion: if the median absolute deviation (MAD) of their power in the 60 Hz or 300 Hz bands exceeded 6 standard deviations from the group mean (assessed over the central 20% of trials), they were removed prior to further processing. Cleaned signals were then re-referenced using a bipolar referencing among channel pairs, approximating neural activity in a 5 mm sphere between the two contacts.

Neural responses were epoched from −1000 to +2000 ms relative to stimulus onset. Stimulus onsets were determined by cross-correlating each stimulus waveform with the simultaneously recorded auxiliary audio trace, allowing us to synchronize neural data to audio with millisecond precision. Each trial was baseline-normalized using a − 900 to −100 ms window, reducing intertrial variability and accounting for ongoing background activity.

We extracted broadband high-frequency activity (BHA; 70–150 Hz) as a physiological proxy for local population-level neuronal firing. (Ray and Maunsell, 2011) BHA is known to correlate closely with multiunit spiking, BOLD fMRI responses, and local dendritic activity, and is sensitive to both bottom-up sensory drive and top-down feedback processes (Crone et al., 2011; Leszczyński et al., 2020) BHA was computed by filtering the data in eight log-spaced sub-bands between 70 and 150 Hz and bandwidths 16 to 64 Hz, using sixth-order zero-phase Butterworth filters applied in both forward and reverse directions to avoid phase distortions. The analytic signal was obtained for each band using the Hilbert transform, and the resulting amplitude envelopes were z-scored relative to the pre-stimulus baseline. To eliminate edge artifacts introduced by filtering, we discarded the first and last 100 ms of each trial segment. The final BHA signal was computed as the mean normalized amplitude across all eight bands, yielding a single broadband BHA time course per trial.

### 5.3. Cortical surface reconstruction and channel localization

High-resolution anatomical localization of iEEG electrode contacts was achieved through multimodal image coregistration, cortical surface reconstruction, and atlas-based labeling. Pre-operative T1-weighted imaging was first processed using SynthSR, a deep convolutional neural network implemented in FreeSurfer that synthesizes 1 mm isotropic MP-RAGE volumes from heterogeneous clinical scans while inpainting lesions and correcting signal distortions (Iglesias et al., 2021; Iglesias et al., 2023). Cortical reconstruction and volumetric segmentation were then performed using the FreeSurfer image analysis suite (http://surfer.nmr.mgh.harvard.edu/), following standard protocols (Dale et al., 1999; Fischl et al., 1999). Post-implantation CT scans were resampled to 1 mm^3^ isotropic resolution using SPM12 and co-registered to each subject’s pre-operative T1-weighted MP-RAGE using the CT2MRI rigid-body coregistration algorithm (https://github.com/ajoshiusc/USCCleveland/tree/master/ct2mrireg). iEEG contact centroids were manually labeled on the co-registered CT in Brainstorm (version 23-Sep-2024, implemented in MATLAB R2022a). Anatomical labels for each electrode were derived using the Human Connectome Project (HCP) multimodal parcellation atlas, extended version 1.1 (Huang et al., 2022). The volumetric atlas was resliced to 1 mm^3^ resolution and transformed into the subject’s native space using the inverse deformation field. For each contact pair, a 5 mm spherical search volume (524 mm^3^) was placed at the midpoint between electrode coordinates, and the most prevalent voxel label within this sphere was assigned to the channel pair. This approach enabled robust assignment of electrode contacts to cortical regions while accounting for minor coregistration uncertainty and anatomical variability.

### 5.4. Lesion mapping

The extent of resection that the patient presented with at 9 years of age was determined by generating binary ROI masks for each parcel of the HCPvolumetric atlas, (Huang et al., 2022; Huang et al., 2021)which were applied to the reconstituted, filled-in T1 image volume (from SynthSr) and original pre-iEEG T1 using the Nilearn library in Python (Research Resource Identifier: RRID:SCR_001362). Masked image volumes were binarized using Otsu’s method for grayscale image thresholding (Otsu, 1979) and subtracted from one another. The count of non-zero voxels in the output volume was divided by the total number of non-zero voxels in the synthetized T1 to estimate the extent of resection in each ROI.

### 5.5. Fiber tracking and structural connectivity

Diffusion-weighted images were acquired on a Siemens Prisma scanner using an axial EPI diffusion sequence (DTI-30; TE = 95 ms, TR = 8000 ms). Images were collected with 2-mm slice thickness, 2.6-mm interslice spacing, an in-plane resolution of 1.95 × 1.95 mm, a 90° flip angle, and a pixel bandwidth of 1565 Hz. Thirty diffusion directions were acquired using a b-value of 1000 s/mm^2^. All images were resampled to 2.0-mm isotropic resolution prior to reconstruction.

Diffusion data were corrected for eddy current–induced distortions using FSL eddy. Restricted diffusion was quantified using restricted diffusion imaging (Yeh et al., 2017), and fiber orientation distributions were reconstructed using generalized q-sampling imaging (Yeh et al., 2010) with a diffusion sampling length ratio of 1.25. Tensor-derived diffusion metrics were computed using diffusion-weighted images with b-values < 1750 s/mm^2^.

Deterministic tractography was performed using a quantitative anisotropy–based tracking algorithm11 with augmented tracking strategies (Yeh, 2020) to improve reproducibility. Seed regions were placed at the stimulation sites in left pmHG and pSTG. Tracking parameters were randomized across streamlines, with anisotropy thresholds sampled between 0.5 and 0.7 times the Otsu threshold and angular thresholds sampled between 45° and 90° Streamlines shorter than 30 mm or longer than 200 mm were excluded. A total of 1000,000 seeds were initiated. Shape analysis43 was used to derive tract-level metrics, and connectivity matrices were constructed based on the quantitative anisotropy of streamlines connecting HCPex parcellation regions. All diffusion analyses were performed using DSI Studio (http://dsi-studio.labsolver.org).

### 5.6. Mapping categorical responses to vocal sounds

Auditory stimuli were modified versions of the Belin Voice Localizer (Belin et al., 2000). These original stimuli were designed for fMRI experiments and consisted of 8-second clips, with each clip containing a series of either vocal (e.g., speech, laughter, and coughing) or nonvocal (e.g., mechanical, music, and animal vocalizations) sounds only. As described in prior work(Rupp et al., 2022),these stimuli were adapted to capitalize on the temporal resolution afforded by intracranial recordings: PRAAT silence-based segmentation was used to extract and save individual sounds from each clip. Sounds with duration shorter than 550 ms were discarded; all other sounds were shortened to this duration, linear-ramped on and off by 50 ms, and RMS-normalized. This procedure generated 80 nonvocal and 72 vocal sounds; to ensure balanced classes, only the first 72 nonvocal sounds were included.

Stimuli were presented binaurally via Etymotic ER-3C earphones, with volume adjusted to a “loud, but comfortable” level for the patient before the start of the experiment. Inter-stimulus intervals were randomized uniformly between 1 and 2 s. The patient was instructed to indicate 1-back stimulus repeats using a button box (Response Time Box, v6). Repeats occurred on 16% of all trials. Each stimulus was presented a minimum of 2 times, although the 1-back task design resulted in some stimuli with more presentations (up to 4 maximum).

As described in prior work,4 time-varying two-sample t-statistics were calculated for every channel BHA of single-trial responses (10,000 permutations). The magnitude of difference between BHA to vocal and non-vocal stimuli was quantified as the sum cluster mass (i.e., the sum of masses for all significant (*p* < 0.001) clusters for a given channel). Channels that showed significant BHA responses during the sound presentation window relative to baseline without V–NV separability were determined to be “auditory”, while channels that were both auditory responsive and showed V–NV separability were labeled as “voice selective”. Sites without any significant task-evoked activity were nominated as candidates for positive control stimulation sites.

### 5.7. Behavioral-Stimulation experiment

We designed a two-alternative forced choice task in which the participant judged whether a natural sound was a vocal or nonvocal sound. The same set of natural sounds was used as described in the auditory 1-back memory task, but stimuli were only presented once each (total *n* = 144). The patient used the button box to select “Vocal” or “Nonvocal” for each stimulus presentation.

During presentation of the auditory stimulus, we delivered bipolar electrocortical stimulation (ECS) between channel pairs, separately and in blocks, at three regions of interest: left pmHG, left pSTG, and a positive control in left SFS. Compared to other control site candidates, the site in SFS was determined by the clinical team to carry a low risk of after discharge. A negative control (sham) block was also used, during which no stimulation was delivered. ECS had the following parameters: 2 mA, 1 Hz, biphasic, bipolar 25 ms square pulse, delivered in trains of 5 pulses each, with 5–6 s in between trains. The start of each train was timed to roughly coincide with the presentation of auditory stimulus. In total, 38 trains were delivered to pmHG, 36 trains were delivered to pSTG, and 39 trains were delivered to SFS.

### 5.8. Trial-wise response onset calculation

We estimated trial-wise onset latencies following prior work (Coon and Schalk, 2016). For each channel and trial, BHA was z-scored to the pre-stimulus baseline (e.g., −500 to −100 ms), then restricted to the task window (e.g., 0–550 ms). For each retained channel, we swept a range of z-score thresholds and selected the value that maximized the difference in supra-threshold sample counts between task and baseline segments (normalized by their durations). Onsets were then defined per trial as the first time point in the task window at which the absolute z-score exceeded this threshold for at least 50 ms.

### 5.9. Cortico-cortical evoked potential (CCEP) analysis

We took advantage of the low frequency stimulation protocol to probe functional connectivity between the stimulated regions via CCEPs. CCEPs measure not only functional connectivity but also directionality between stimulation and recording sites (Kobayashi et al., 2024) and are well-validated in auditory and language pathways (Enatsu et al., 2016; Matsumoto et al., 2004; Ookawa et al., 2017) There were six stimulation/recording conditions to consider, which we designated as “stimulation site → recording site”: pmHG → pSTG; pmHG → SFS; pSTG → pmHG; pSTG → SFS; SFS → pmHG; and SFS → pSTG.

Similar to the procedure for extraction of BHA, iEEG recordings from the stimulation experiment were first bipolar re-referenced to their adjacent electrode to better approximate local neural activity remove any noise or stimulation artifacts that were common to adjacent channel pairs. The re-referenced signals were then high-pass filtered using a 1-Hz cutoff (1st-order, forward/reverse). Each trial was subsequently centered on the stimulation pulse at *t* = 0, with an epoch window spanning 500 ms pre-stimulation to 950 ms post-stimulation. Because the start of each train of five pulses coincided with stimulus presentation, our analysis of CCEPs included only those evoked by the fourth and fifth pulses to mitigate any contamination from auditory LFPs. Trial data were baseline corrected by subtracting the channel’s median of baseline activity from each trial, calculated 500 ms to 50 ms pre-stimulation.

Trials were analyzed for artifacts via CRP (Miller et al., 2023) over the window *t* = 15 ms to *t* = 900 ms post stimulation. The first 15 ms were excluded to mitigate any stimulation artifact contamination that could not be removed by bipolar re-referencing alone. Intertrial reliability was used to reject artifactual trials that fell below the mean projection magnitude of the other trials. We hypothesized that single trials would be artifactual if their time course was significantly different from the others with a difference threshold quantified at *p* < 10^−6^. As suggested in prior work, (Miller et al., 2023) we iterated through this process using CRP for each stimulation/recording pair until no artifacts remained below this threshold. After procedural artifact rejection, CRP was again used to determine both general and trial-wise features for each of the six stimulation/recording pairs. General features included extraction significance, which uses intertrial reliability to measure significance of a response, and a canonical response, *C(t)*, waveform unique to that stimulation/recording pair derived from the principal component analysis of its trials (PCA). *C(t)* is comparable to grand averaging commonly used in neurophysiology studies; however, PCA better preserves trial-to-trial variance. *C(t)* duration is constrained to the time by which it ceases to be reliable after stimulation, which we refer to as the duration of the canonical response.

Extraction significance was determined by a 1-sample *t*-test (testing a stimulation/recording pair’s mean projection magnitude against a null response). To correct for false discovery rate (FDR), we used the Benjamini-Yekutieli procedure for each of the six stimulation/recording pairs, as outlined in prior work, where p-values below 0.001 were considered significant (Ojeda Valencia et al., 2023) . Six-way nonparametric ANOVA (Kruskal-Wallis) was conducted on the sample distributions of each pair’s parameters (alpha prime – the weight of the C(t) in a given trial over the duration of the canonical response, and signal-to-noise ratio (SNR), quantified as the ratio of alpha prime and the magnitude of the residual). Dunn’s post-hoc tests were conducted on sample distributions and Bonferroni-corrected p-values below 0.05 were considered significant.

For the supplemental CCEP analysis, we parameterized responses to stimulation in valid channel pairs for each of the three stimulation conditions. Channel pairs were excluded from a condition if they were the channel pair being stimulated or contained a channel that was part of the stimulation pair. Channels were also excluded if they exhibited extreme spikes that could not be removed with bipolar re-referencing (often these spikes were >10 standard deviations from baseline), or if one or both channels in a pair were in either white matter or outside the brain. This resulted in analysis of 105 channel pairs during stimulation of pmHG, 104 channel pairs during stimulation of pSTG, and 101 channel pairs during stimulation of SFS.

We used the same procedure described above for artifact rejection, then used CRP to obtain alpha prime and SNRs for each channel pair, and visualized approximate channel pair locations on an inflated brain surface using FreeSurfer.

### 5.10. Time-domain multivariate Granger causality (MVGC) analysis

MVGC was estimated using the MVGC toolbox (Barnett and Seth, 2015) on auditory ERPs during the 1-back task. ERPs were resampled to 100 Hz, bipolar re-referenced to the adjacent electrode, and baseline corrected by subtracting the mean pre-stimulus period (*t* = −1000 to 0 ms). The Akaike Information Criterion (AIC) was used to estimate a best model order of 2. Because we wanted our model to be conditioned on as many pertinent variables as feasible, pairwise time-domain Granger F-statistics were computed for all valid bipolar re-referenced channel pairs (*n* = 108) during the poststimulation period (*t* = 0–1000 ms). For each directed channel pair (pair A → pair B), the null hypothesis was that inclusion of lagged values of A does not improve prediction of B beyond a model including only lagged values of B. Statistical significance across channel pairs was assessed using an FDR-corrected alpha of 0.001. Validity criteria for channel pair inclusion were the same as those for CCEP analysis, but because no stimulation was delivered during the 1-back task, we were able to include those channel pairs which had been stimulated in the separate CCEP experiment. These criteria allowed us to determine whether CCEPs and MVGC could supply converging evidence for functional connectivity at the level of the channel pair.

## Abbreviations:

iEEG: Intracranial-electroencephalography
ROI: Region of interest
pmHG: Posteromedial Heschl’s gyrus
pSTG: Posterior superior temporal gyrus
SFS: Superior frontal sulcus
VL: Voice localizer
V/NV: Voice/nonvoice
CVP: Categorical voice perception
BHA: Broadband high-gamma activity
CCEP: Cortico-cortical evoked potential
CRP: Canonical response parametrization
